# Electrical Properties of Laser Patterned Schottky Diode with ALD-Grown TiO_2_ Interlayer

**DOI:** 10.1021/acsomega.4c01585

**Published:** 2024-05-02

**Authors:** Elanur Dikicioǧlu, M. Burcu Balı, Semran Saǧlam, Halil Berberoǧlu, Ihor Pavlov, Arian Goodarzi, Elif Orhan

**Affiliations:** 1 Vocational School of Health Services, Yüksek İhtisas University, 06291 Ankara, Turkey; 2 Faculty of Science, Department of Physics, Gazi University, 06500 Ankara, Turkey; 3 Physics, Ankara Hacı Bayram Veli University, 06570 Ankara, Turkey; 4 Department of Physics, Middle East Technical University, 06800 Ankara, Turkey; 5 ODTU-GUNAM, Center for solar energy research and application, 06800 Ankara, Turkey

## Abstract

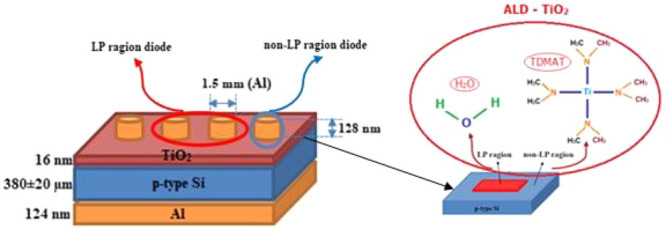

Heterojunction formation is the key to adjusting the electronic and optoelectronic properties of various semiconductor devices. There have been various reports on the formation and importance of semiconducting heterojunction devices based on metal oxides. Titanium dioxide (TiO_2_) is one of the metal oxides that has many unique properties. TiO_2_’s importance is due to its physical and chemical properties such as large band gap, large permittivity, stability, and low leakage current density. In this context, we present the electrical properties of the metal–insulator–semiconductor (MIS) type-TiO_2_-based Schottky barrier diode (SBD) in the study. To create a thin layer of TiO_2_ on *p-type silicon* (*p-type* Si) patterned partially by the laser-induced periodic surface structure (LIPSS) technique, an atomic layer deposition (ALD) technique was used in the study. For comparison, the current–voltage (*I*–*V*) characteristics of the TiO_2_-based laser-patterned (LP) and nonlaser-patterned (non-LP) diodes were measured at 300 K and in the dark at ±5 V. Classical thermionic emission (TE) theory and Cheung functions were used to investigate the critical diode parameters of the diodes, including ideality factor (*n*), series resistance (*R*_s_), and barrier height (Φ_b_). The *n* values were obtained as 4.10 and 3.68 from the TE method and Cheung functions for the LP diode, respectively. The Φ_b_ values were found as 0.68 and 0.69 eV from the TE method and Cheung functions, respectively. According to experimental results, the laser patterning resulted in an increase in the Φ_b_ values and a decrease in the *n* values. After laser patterning, it was observed that the device worked effectively, and the ideality factor and barrier height values were improved. This study provides insight into the fabrication and electrical properties of TiO_2_-based heterojunction devices.

## 1 Introduction

A semiconductor device called a Schottky diode is created when a metal and a semiconductor come together. This diode generates an energy barrier using a metal–semiconductor junction. Schottky diodes are dependent on the energy barrier that develops at the interface between a semiconductor and a metal. Because of their quick switching speed and limited reverse voltage tolerance, diodes are frequently used in high-frequency applications. They also display nonlinear voltage–current characteristics. Metals, insulators, and semiconductors are all combined to form metal–insulator–semiconductor (MIS) structures. By adjusting the electric field, this arrangement may regulate the carrier density on the semiconductor surface. MIS structures are frequently seen as components with a capacitance. The semiconductor surface is connected to the insulator that sits between the metal electrode and the semiconductor. Applying an electric field has an impact on the carriers in semiconductors. MIS structure is frequently seen in capacitive devices and semiconductor memory devices, and it is utilized as an isolation layer in transistors and integrated circuits. The insulator layer in the metal–insulator sandwich structures is usually bonded directly to a semiconductor surface and can be made of several materials, not just oxide. An oxide material such as SiO_2_, Al_2_O_3_, ZnO, or TiO_2_ is commonly employed as the insulator layer in metal–oxide–semiconductor (MOS) systems.^1,2^ Particularly in MOS transistors and integrated circuits, these structures are commonly used.

The formation of heterojunctions is the key to tuning the electronic and optoelectronic properties of various semiconductor devices. The formation and importance of semiconducting heterojunction devices based on metal oxides have been widely reported.^3−5^ TiO_2_ is a high-dielectric, high-refractive, big band gap material that is widely employed in many different electronic devices, especially in the memory, photovoltaic, and optical coatings industries.^6,7^ It is being investigated in several papers for application at the MS interface to provide barrier modification in addition to the semiconductor layer’s surface passivation. Before Schottky metallization, a TiO_2_ thin-film layer deposition provides several technological benefits, including enhanced device impedance characteristics and shielding the semiconductor surface from defective interfacial compounds.^8,9^ In other words, because of potential interfacial reactions resulting from the deposition of a rectifying metal contact, TiO_2_ can be added to the MS interface to control the defective states. Consequently, efforts are focused on improving the rectification capabilities and increasing the junction barrier in these kinds of diodes.^10,11^

Atomic layer deposition (ALD) is one of the most viable ways to successfully create TiO_2_ in diode applications, even if there are other methods used to deposit TiO_2_ thin-film layer at the MS interface, such as vapor deposition and solution-based techniques.^12−17^ It can produce dense, smooth films with fewer flaws and controllability over the film thickness when utilized in the deposition of TiO_2_ layers. Aydın et al.^18^ investigated the electrical analysis of the Al/p-Si Schottky diode with TiO_2_ thin film performed at room temperature. They concluded that the Schottky effect was found to be dominant in the reverse direction. A researcher^19^ studied the diode parameters of the Al/TiO_2_/p-Si Schottky diode at a wide temperature range and different illuminance intensities. With the increasing temperature, the ideality factor value of the device decreases from 4.878 to 2.305 and the barrier height increases from 0.287 to 0.714 eV. The electrical characterization of the Al/ALD-grown TiO_2_/p-Si structure was investigated by Karabulut et al.^20^ The optoelectronic properties of Li:TiO_2_-based photodiodes were investigated.^21^ It was concluded that the lowest *n* value of 5.92 is obtained for a 2% Li-doped diode. The *I*–*V* measurements of Al/TiO_2_/*n*-Si were taken in the temperature range of 50 K–400 K.^22^ The results showed that the currents of the devices are a strong function of the temperature. Yıldız et al. fabricated the Al/TiO_2_/p-Si Schottky-type by atomic layer deposition (ALD) for a thin layer and investigated *I*–*V*–*T* properties.^23^ Boutelala et al.^24^ obtained the characteristic parameters of Al/TiO_2_/p-Si/Al from *I*–*V* measurement. It was concluded that the diode exhibits perfect photosensitivity and high photoresponse properties. Additionally, TiO_2_ is typically utilized in the UV light detection spectrum. TiO_2_ nanorods were produced using the hydrothermal technique by Gao et al.^25^ Nicolaescu et al.^26^ created a heterostructured p–n type photodetector with high responsivity performance by thermal oxidation of n-TiO_2_ and using p-CuMnO_2_ to get n-TiO_2_/p-CuMnO_2_ thin film. TiO_2_ is occasionally employed as an interfacial layer to improve the photodetection capabilities of Schottky-type photodetectors in the presence of sunlight.^27−30^ The device performed well in the UV and visible ranges if TiO_2_ is placed on the Si substrate to create a photodetector.^31^ The effect of laser patterning (LP) on Schottky diode properties and graphene film quality was investigated by Orhan et al.^32^ They showed that laser patterning reduces reverse-biased leakage currents and increases the Schottky barrier height. In particular, they have seen how the laser patterning process affects 3D graphene nanosheets, resulting in the formation of new structures over the entire surface of the p-type substrate. These structures were in the form of nanospheres and nanoroses. According to their results, it is expected that the ideality factor and the barrier height will be improved after the laser patterning structuring and that the device will work effectively.

This study is focused on investigating the effect of the LP process on the diode properties of *ALD-grown* TiO_2_/p-type Si Schottky structures. By comparison of two diodes consisting of oxide (TiO_2_) junctions with an LP diode and a non-LP diode at 300 K in the dark, the effect of LP on the TiO_2_-p-type Si interface was evaluated. In this study, only the central region of the p-type Si wafer was patterned by LIPSS. The TiO_2_ layer was deposited directly on p-type Si with patterned and nonpatterned regions by the ALD technique for the fabrication of Al/*ALD-grown* TiO_2_/p-type Si Schottky diodes. Current–voltage (*I*–*V*) characteristics were measured in the dark at 300 K at ±5 V. Using the methods of Cheung and the standard TE theory, the ideality factor (*n*), the barrier height (ϕ_*b*_), and the series resistance (*R*_s_) of the fabricated diodes were extracted.

## 2 Experimental Section

**Figure 1:**
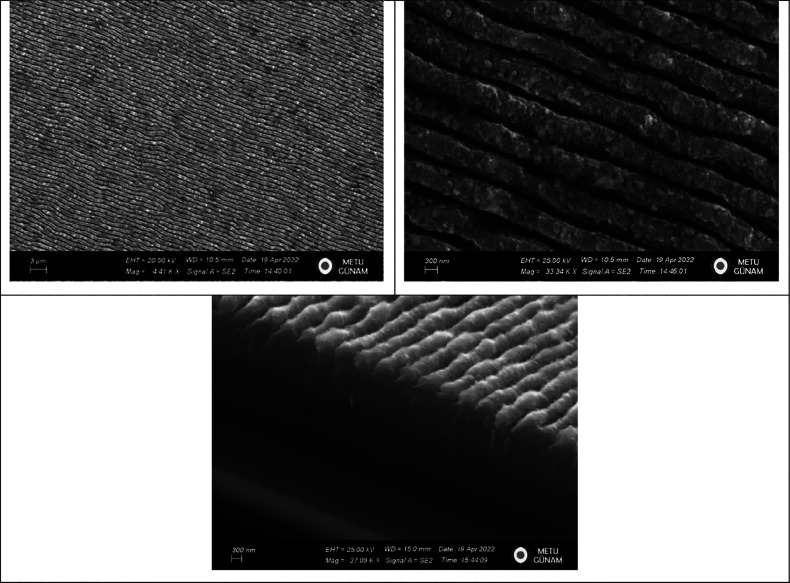
SEM images of laser-patterned *p-type Si* wafer.

In this work, we employed a *p-type Si* (100) wafer with a resistivity of 1–10 Ω·cm and a thickness of 380 μm. The *p-type Si* wafer was cleaned using Radio Corporation of America (RCA) techniques to get rid of any organic residues before processing.^33^ The central region of *p-type Si* was patterned by using a femtosecond laser source. A central region of *p-type* Si was patterned by only the LIPSS technique, but the whole wafer was coated with a TiO_2_ layer by the ALD technique. The laser source (up to 2 μJ pulse energy; ∼300 fs pulse duration) was a homemade fs-fiber laser with a 1 MHz rep rate at 1030 nm. After the laser was focused on the Si substrate, a Galvo scanner was used to raster-scan the area, which measured 8 × 8 mm^2^. On Si, the beam diameter was about ∼9 μm. The overlap factor was 5–6 pulses per spot, and the surface pulse energy was 400 nJ. A scanning electron microscopy (SEM) images of the laser patterned *p-type Si* wafer are shown in Figure 1. As can be seen in Figure 1, this results in a regular periodic path. In this study, two diodes with TiO_2_ layer laser pattern (LP) and nonlaser pattern (non-LP) were created. The *I*–*V* characteristics of the two diodes were measured in the ±5 V range at 300 K and in the dark. Using sputtering systems operating at 500 °C and 4.7 × 10^–6^ Torr pressure, 128 nm thick Al (99.999%) metal was formed to create an ohmic contact on the unpolished back surface of the plate. An ALD device (Okyay Nanotech) was used to deposit a TiO_2_ layer as a 16 nm thin film on the entire surface of the *p-type Si* wafer, including both laser-patterned and nonlaser-patterned regions. This was carried out at 170 °C, a low substrate temperature. To reach a layer thickness of 16 nm at a growth rate of 1.29 Å/cycle, 125 cycles of TiO_2_ deposition were carried out. Tetrakis (dimethylamido) titanium(IV) (TDMAT) and H_2_O were used as precursor materials of TiO_2_. To separate precursor cycles, nitrogen (%99.999) was employed as a cleaning gas and carrier at a flow rate of 7 sscm. The percussive temperature is 150 °C; the circle inner temperature is 170 °C, and the system pressure is 2.72 × 10^–1^ Torr. A 100 ms TDMAT pulse, a 20 s N_2_ purge, a 15 ms H_2_O pulse, and a 10 s N_2_ purge make up a single TiO_2_ ALD cycle. Subsequently, using a mask shaped like 1.5 mm circular dots, 128 nm thick Al (∼99.999%) rectifier contacts were formed on the TiO_2_ layer using the sputtering process at 7.06 × 10^–6^ Torr.

**Figure 2:**
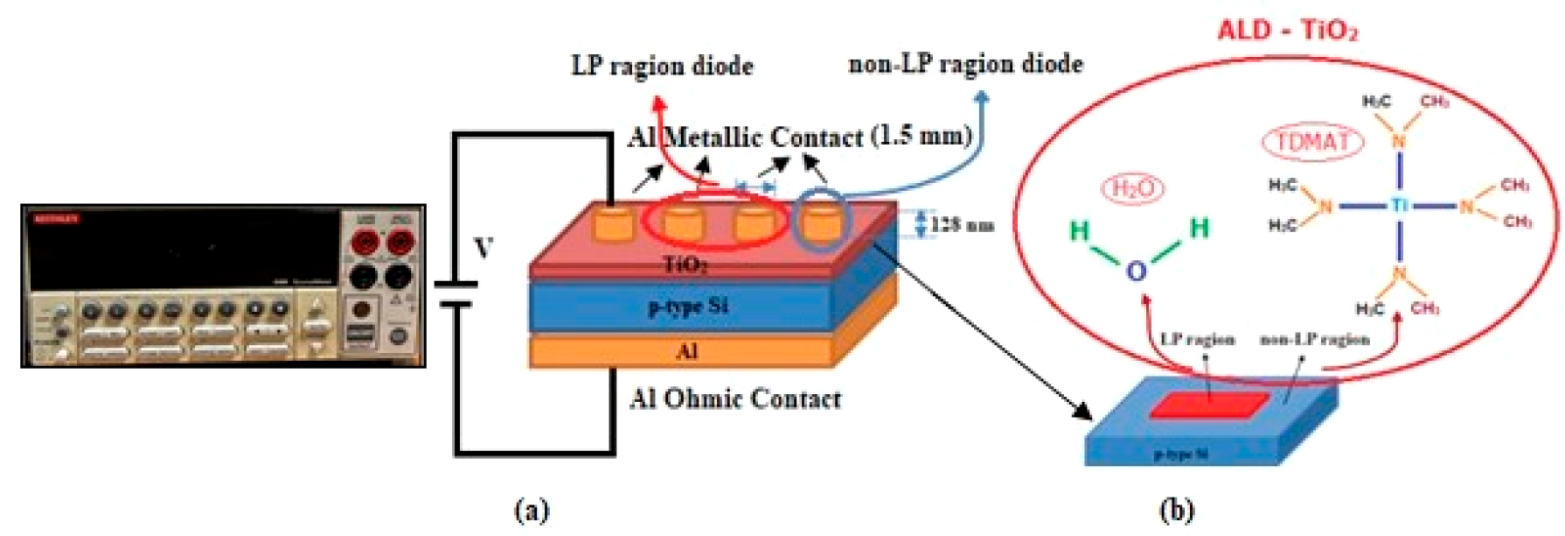
(a) Measurement setup. (b) Schematic sections of Al/*ALD-grown TiO*_2_/p-type Si for LP (central region) and non-LP diode (edge region).

Figure 2 displays the measurement setup and schematic cross-section of the Al/*ALD-grown* TiO_2_/p-type Si diode. Keithley 2400 programmable constant current source was used for current–voltage (*I*–*V*) measurements. All of these measurements were carried out by controlling the device with the help of an IEEE-488 AC/DC converter card plugged into the computer. All of the electrical data presented in the study was collected from both the LP diode and the non-LP diode. In this study, only the central region of the p-type Si wafer was patterned by LIPSS. The TiO_2_ layer was deposited directly over the entire region, including patterned and nonpatterned regions, using the ALD technique to fabricate Al/*ALD-grown* TiO_2/_p-type Si Schottky diodes on p-type Si.

**Figure 3:**
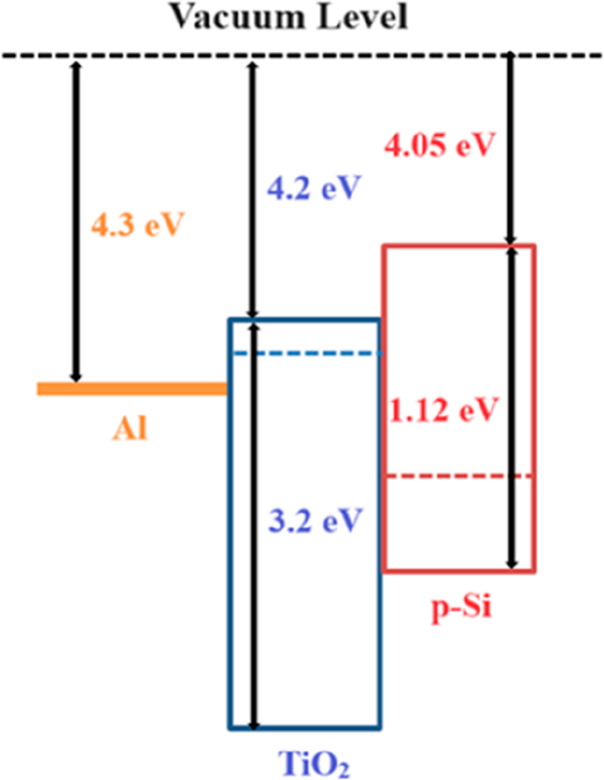
Energy band diagram of Al/*ALD-grown TiO*_2_/*p-type Si* diode.

Figure 3 shows the energy band diagram for the Al/*TiO*_2_/*p-type Si* diode. The electron affinities of p-Si, anatase phase TiO_2_, and l metal have been reported to be approximately 4.05,^34^ 4.2,^18,35^ and 4.3 eV,^36^ respectively.

## 3 Results and Discussion

First, the TE theory has been used to analyze the electrical properties of the Al/*ALD-grown TiO*_2_/*p-type Si* diode. The relationship between the current flowing through a Schottky diode and the forward bias voltage (*V* > 3*kT*/*q*) is as follows, for the TE theory.^37^
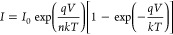
1
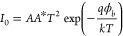
2where *I*_0_ is the saturation current found at *V* = 0 at the (ln *I*)–*V* plot’s straight-line intercept. A* is Richardson’s constant (32 A/(cm^2^ K^2^) for *p-type* Si). The electron charge, Boltzmann’s constant, and temperature in Kelvin are denoted by the letters *q*, *k*, and *T*. The diode area, effective barrier height, and applied voltage are denoted by the letters *A*, ϕ_b_, and *V*, respectively.

**Figure 4:**
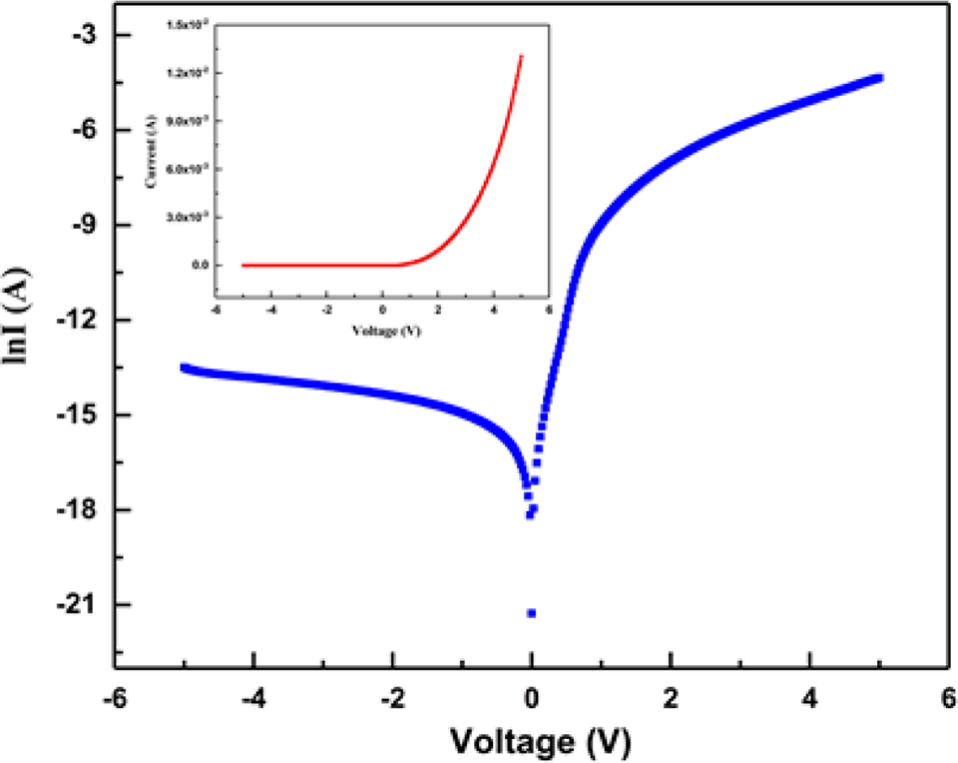
Semilogarithmic *I*–*V* characteristics of the Al/ *ALD-grown TiO*_2_/*p-type Si* diode.

Using eq 3, the slope of the semilogarithmic *I*–*V* plots for the linear region was used to get the values of *n*.^37^
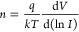
3ϕ_b_ can be derived from eq 4 as given by
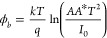
4Figure 4 shows the (ln *I*)–*V* characteristics of an Al/*ALD-grown TiO*_2_/*p-type Si* diode at ±5 V and 300 K. The diode’s *I*–*V* curve is displayed in the embedded graph in Figure 4. ϕ_b_ values were calculated from the intercepts of the forward bias (ln *I*)–*V* plot at 300 K.

**Figure 5:**
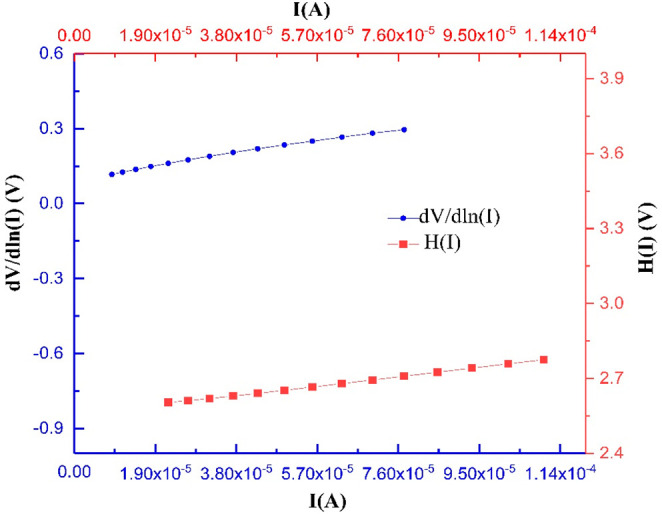
d*V*/d(ln *I*) vs *I* and *H*(*I*) vs *I* characteristics of Al *ALD-grown TiO*_2_/*p-type* Si diode.

Figure 4 makes it clear that despite low leakage, the *I*–*V* characteristics of the Al/*ALD-grown TiO*_2_/*p-type Si* structure exhibit rectifying behavior. The diode’s rectification ratio (RR) was found to be 9.5 × 10^3^. In the linear region, which is the forward bias *I*–*V* characteristic downward curvature, *R*_s_ is considerable. But in both the nonlinear and linear regions of *I*–*V* characteristics, the other two factors, *n* and ϕ_b_, are important. A method created by S.K. Cheung and N.W. Cheung was used to calculate the values of *n*, ϕ_b_, and *R*_s_.^37^ For a nonlinear high-voltage region of the forward bias of *I*–*V* plots, Cheung’s model is implemented.^38^ According to this method, *n*, ϕ_b_, *R*_s_, and *H*(*I*) can be written as
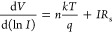
5
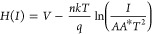
6
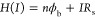
7The ϕ_b_ was obtained from the linear region in the forward bias *I*–*V* characteristics. In Figure 5, the d*V/*d ln *I* vs *I* and *H*(*I*) vs *I* plots are presented for an Al/*ALD-grown TiO*_2_/*p-type* Si diode at 300 K, respectively.

The slope of the d*V*/d(ln *I*) vs *I* curve yields the value of *R*_s_, while the curve’s cut-point yields the value of *n*. Using the *n* value in eq 5, *R*_s_ is obtained from the slope of the *H*(*I*) vs *I* curve and ϕ_b_ values are obtained from the intersection point of the curve in eq 7. The values of *n*, ϕ_b_, and *R*_s_ for the Schottky diode are computed, as shown in Table 1. For Al/*ALD-grown TiO*_2_/*p-type* Si, the diode parameters for the LP and non-LP regions are given in Table 1.

**Table 1:** Diode Parameters for the LP Diode and the Non-LP Diode

		LP diode			Non-LP diode		
Methods		*n*	Φ_b_ (eV)	*R*_s_ (kΩ)	*n*	Φ_b_ (eV)	*R*_s_ (kΩ)
TE		4.10	0.68	-	8.13	0.51	
Cheung	d*V*/d ln *I* vs *I*	3.68	-	2.99	4.97	-	0.80
	*H*(*I*) vs *I*	-	0.69	1.93	-	0.38	0.73

It was found that the *n* values were obtained as 4.10 and 3.68 from the TE method and Cheung functions for the LP diode, respectively. The Φ_b_ values were found as 0.68 and 0.69 eV from the TE method and Cheung functions, respectively. For the non-LP diode, the *n* values were obtained as 8.13 and 4.97 from the TE method and Cheung functions, respectively. The Φ_b_ values were found as 0.51 and 0.38 eV from the TE method and Cheung functions for the non-LP region, respectively. The results in Table 1 show a good agreement (1% discrepancy) between the barrier height (Φ_b_) values obtained by the TE and Cheung methods. This confirms the effectiveness of these techniques. However, there is a discrepancy of about 10% between the nanovalues obtained by the two methods for the LP region. An increase in *R*_s_ values for the LP region is attributed to the defects and surface roughness created on p-type Si by laser patterning. The nonideal behavior (*n* > 1) of the diode confirmed the presence of interfacial states. The Φ_b_ values for all two methods are in agreement with each other for the LP-diode.

The periodicity of LIPSS on silicon for the laser used (1030 nm) is around 850–900 nm. Therefore, this periodicity is greater than the thickness of the TiO_2_ layer (16 nm) and the metal contact (128 nm) and it can be said that the layers will take the shape of the pattern during deposition. As a result, the effective contact surface of metal contacts is expected to increase compared with the flat surface. Laser patterning resulted in an increase in the Φ_b_ values and a decrease in the *n* values. After laser patterning, it was observed that the device worked effectively and the ideality factor and barrier height values were improved. Therefore, this effect may confirm an improvement in the functioning of the device. For comparison with our work, we have presented the diode parameters of various Schottky diodes with TiO_2_ interlayers of different thicknesses fabricated by some researchers^18−24^ at 300 K in the literature in Table 2.

**Table 2:** Comparison of Diode Parameters for Various Types of Schottky Diodes with TiO_2_ Interlayer

Diodes	Interlayer Coating Methods	*n* (LP diode	Φ_b_ (eV)	RR	References
Al/TiO_2_ (16 nm)/laser-patterned p-Si	ALD	4.10 (TE)	0.68	9.5 × 10^3^	Present work
		3.68 (Cheung)	0.69		
Al/TiO_2_ (200 nm)/p-Si	ALD	2.96 (TE)	0.66	-	(18)
		2.55 (Cheung)	0.76		
Al/TiO_2_ (20.4 nm)/p-Si	Thermal evaporation	2.437	0.68	1.77 × 10^3^	(19)
Al/TiO_2_ (10 nm)/p-Si	ALD	1.04	0.80	-	(20)
Al/TiO_2_/p-Si	Sol–gel spin coating	9.41	0.76	4.75 × 10^3^	(21)
Al/TiO_2_ (200 nm)/n-Si	Spin coating	1.93 (TE)	0.93	2.49 × 10^4^	(22)
		2.26 (Cheung)	0.84		
Al/TiO_2_ (4 nm)/p-Si	ALD	12.71 (TE)	0.64	-	(23)
		12.73 (Cheung)	0.68		
Al/bilayer TiO_2_ (226.92 nm_)_/p-Si	Spin coating	1.91	0.71	3 × 10^4^	(24)

As can be seen from Table 2, the thickness of the interlayer and the difference in the method used to create the measurements affected the *n* and Φ_b_ values. When the basic diode parameters of the device created in this study were compared with other studies, especially *ALD-grown* structures [refs (18), (20), and (23)], we found a high *n* value (8.13) for the non-LP diode as in ref (23) (see Table 1). The height of the barrier was found to be compatible with the study mentioned above at 6%.^23^ After laser pattering, the *n* value (calculated by the TE method) of the LP diode decreased by a factor of 50%. Compared to the structure grown by ALD by the researchers,^20^ the *n* values for the LP diode are higher than that of the work. As no RR data exist for the *ALD-grown* TiO_2_ structure, it could not be compared. The RR was higher than those of thermally evaporated^19^ and sol–gel spin-coated^21^ structures. A high RR value was obtained for the spin-coated bilayer TiO_2_ structure^22^ as shown in Table 2.

## 4 Conclusion

In this study, the electrical properties of the Al/*ALD-grown TiO*_2_/*p-type* Si Schottky barrier diode fabricated in the form of a heterojunction were examined in the dark and at 300 K using TE theory and Cheung methods. To create a thin layer of TiO_2_ on both the LP region and the non-LP region on the *p-type* Si substrate, the ALD technique was used in the study. The *I*–*V* characteristics of the diode were investigated. The presence of interfacial states was confirmed by the nonideal behavior (*n* > 1) of the measured values. It can be said that the layers will take the shape of the pattern during deposition. Thus, laser patterning increased the effective contact surface of metal contacts compared to the flat surface, causing an increase in the Φ_b_ values and a decrease in the *n* values. After laser patterning, it was observed that the device worked effectively and the ideality factor and barrier height values were improved. Therefore, this effect may confirm the improvement in the functioning of the device. This study provides insight into the fabrication and the electrical properties of heterojunction devices based on TiO_2_.

## Data Availability

The data that support the findings of this study are available from the corresponding author, [E.O.], upon reasonable request as it is unpublished data for another study.
